# Role of Bruton's tyrosine kinase (BTK) in growth and metastasis of INA6 myeloma cells

**DOI:** 10.1038/bcj.2014.54

**Published:** 2014-08-01

**Authors:** R Bam, S U Venkateshaiah, S Khan, W Ling, S S Randal, X Li, Q Zhang, F van Rhee, B Barlogie, J Epstein, S Yaccoby

**Affiliations:** 1 Myeloma Institute for Research and Therapy, University of Arkansas for Medical Sciences, Little Rock, AR, USA

## Abstract

Bruton's tyrosine kinase (BTK) and the chemokine receptor CXCR4 are linked in various hematologic malignancies. The aim of the study was to understand the role of BTK in myeloma cell growth and metastasis using the stably BTK knockdown luciferase-expressing INA6 myeloma line. BTK knockdown had reduced adhesion to stroma and migration of myeloma cells toward stromal cell-derived factor-1. BTK knockdown had no effect on short-term *in vitro* growth of myeloma cells, although clonogenicity was inhibited and myeloma cell growth was promoted in coculture with osteoclasts. In severe combined immunodeficient-rab mice with contralaterally implanted pieces of bones, BTK knockdown in myeloma cells promoted their proliferation and growth in the primary bone but suppressed metastasis to the contralateral bone. BTK knockdown myeloma cells had altered the expression of genes associated with adhesion and proliferation and increased mammalian target of rapamycin signaling. In 176 paired clinical samples, *BTK* and *CXCR4* expression was lower in myeloma cells purified from a focal lesion than from a random site. BTK expression in random-site samples was correlated with proportions of myeloma cells expressing cell surface CXCR4. Our findings highlight intratumoral heterogeneity of myeloma cells in the bone marrow microenvironment and suggest that BTK is involved in determining proliferative, quiescent or metastatic phenotypes of myeloma cells.

## Introduction

Cumulative evidence indicates that multiple myeloma (MM) emerges from its precursor disease, MGUS (monoclonal gammopathy of undetermined significance), and that alterations both in tumor cells and in their microenvironment likely mediate the conversion from MGUS and asymptomatic MM to overt, symptomatic MM.^1, 2, 3, 4^ In most cases, early-stage disease has MM cells within the interstitial bone marrow (BM) and active disease is characterized by the establishment of a MM niche in the form of focal growth that frequently converts to osteolytic lesions in later stages,^5,6^ depending on molecular properties of the MM cells^7^ and their unique interactions with and dependence on the BM microenvironment.^8,9^ Although MM cells typically grow in BM, most patients with medullary MM also have a small population of circulating MM plasma cells,^10,11^ but the role of these cells in MM metastasis is only partially understood.^9^ The determining factors of MM cell growth patterns—whether dictated by subsets or subclones or by dynamic MM cell plasticity machinery—are under continual investigation, as are the molecular mechanisms by which MM cells home to and metastasize in new BM niches and extramedullary sites.

In addition to various extracellular regulators and their downstream intracellular mediators (for example, protein kinase C, RhoA and RAC1 guanosine triphosphatases),^12,13^ Bruton's tyrosine kinase (BTK) was recently suggested to be involved in mediating MM cell migration and homing to the BM. A nonreceptor tyrosine kinase of the TEC family that is preferentially expressed in hematopoietic cells^14,15^ including MM plasma cells,^16,17^ BTK mediates chemotaxis of MM cells toward stromal cell-derived factor-1 (SDF-1),^16,17^ which is secreted at high levels in the BM. SDF-1 receptor CXCR4 is heterogeneously expressed by a subpopulation of MM cells,^18^ and its presence on the cell surface of primary MM cells highly correlates with *BTK* expression.^16^ This suggests that distinct intraclonal subpopulations of MM cells are involved in tumor-cell adhesion, proliferation and metastasis to new BM niches.

Studies of the direct effects of BTK inhibition on MM cell growth have been inconclusive. The BTK inhibitor, ibrutinib, inhibits MM cell growth *in vitro*, in coculture with osteoclasts or mesenchymal stem cells (MSCs), and in the severe combined immunodeficient (SCID)-hu mouse model for MM.^17^ In contrast, we recently reported that *in vitro* short-term growth of MM cells was not affected by short hairpin RNA (shRNA)-mediated knockdown of BTK or by treatment with BTK inhibitor LFM-A13 and that, in the SCID-rab mouse model, LFM-A13 effectively prevented MM-induced bone disease and insignificantly attenuated tumor growth.^16^ As a single agent, novel BTK inhibitor CC-292 had no anti-MM activity *in vitro* or in animal models but potently inhibited activity of osteoclasts.^19^ Thus, additional studies are needed to unravel the role of BTK in MM cell growth and clonogenicity, particularly within a supportive BM microenvironment.

BTK is not exclusively expressed in MM cells, and the MM BM microenvironment contains numerous hematopoietic cell types; therefore, we examined the consequences of BTK silencing in MM cells on their growth *in vitro* and *in vivo* and on their ability to metastasize to bone in our SCID-rab model for MM.^20^ The study was conducted with the interleukin-6 (IL-6)-dependent INA6 MM cell line. These cells, unlike most MM lines, express high levels of BTK^16,17^ and their growth in SCID-hu or SCID-rab models is restricted to the supportive BM microenvironment.

## Materials and methods

### MM cell line and growth

IL-6-dependent INA6 MM cell line was grown *in vitro* in RPMI-1640 medium (Mediatech, Inc., Manassas, VA, USA) supplemented with IL-6 (R&D Systems, Minneapolis, MN, USA), 10% fetal bovine serum and antibiotics. Authentication of INA6 cells after infection with lentiviral particles was performed using CNV (copy number variant) DNA fingerprinting method developed by Drs Keats and Bergsagel (Mayo Clinic Arizona, Scottsdale, AZ; Keats JJ, personal communication). For *in vivo* metastasis tracking and *in vitro* adhesion assay, INA6 cells were infected with lentivirus containing luciferase/eGFP constructs and quantified with bioluminescence.^21^

Primary MM cells were obtained and processed as described before.^16^

### Methylcellulose colony formation assay

Briefly, INA6 myeloma cells (2 × 10^3^ cells/ml) were suspended in methylcellulose medium (R&D systems) supplemented with 10% serum from myeloma patients, 50 μg/ml penicillin or 50 μg/ml streptomycin or 100 μg/ml Neomycin (Gibco, Grand Island, NY, USA) and 20 ng recombinant human IL-6 as a growth factor for INA6 MM cells. At the end of the 2 weeks, INA6 colonies (>30 cells) were counted under Evos XL core microscope (Life Technologies, Grand Island, NY, USA).

### BTK knockdown and real-time quantitative reverse transcriptase-PCR

shRNA-mediated BTK silencing on INA6 cells and quantitative reverse transcriptase-PCR for MM cells were performed as described previously.^16^

### Adhesion assay

Fibronectin-coated 96-well plates (R&D Systems) were used for adhesion assays. Luciferase-expressing INA6 cells infected with BTK-KD or scrambled control shRNA were seeded at 10^5^ cells/well in 100 μl RPMI medium. After 30 min of incubation, total cells and remaining adherent cells, after nonadherent cells were washed with phosphate-buffered saline (PBS) three times, were measured with bioluminescence assays in Veritas microplate luminometer (Turner Biosystems, Sunnyvale, CA, USA). Percent adhesion was determined from the ratio of bioluminescence of adherent cells to that of total cells. Adhesion assays with fetal MSCs were performed by seeding MSCs in Dulbecco's Modified Eagle's medium at 50% confluence (25 × 10^3^ cells/well) on a fibronectin-coated plate. Plates were washed with PBS three times and INA6 cells seeded as described above.

### Migration and MTT assays

Transwell migration assay and MTT (3-(4,5-dimethylthiazol-2-yl)-2,5-diphenyltetrazolium bromide) growth assay were performed as described before.^16^

### 5-Bromo-2-deoxyuridine labeling index

APC BrdU Flow Kit (BD Biosciences, San Jose, CA, USA) was used to compare cell proliferation. *In vitro* INA6 cells were incubated with 10 μM final concentration of 5-Bromo-2-deoxyuridine (BrdU) for 2 h. Cells were fixed, permeabalized and incorporated BrdU exposed with DNase treatment followed by staining with fluorochrome-conjugated anti-BrdU antibody. BrdU labeling was then measured by flow cytometry method.

*In vivo*, INA6 cells infected with BTK-KD or scrambled control were injected into implanted bones of SCID-rab mice. After 5 and 7 weeks, mice were injected intraperitoneally with BrdU solution (100 μl of 10 mg/ml stock). Two hours later, mice were killed, BM (containing >90% MM cells) flushed and washed with PBS. BrdU labeling was then assessed by flow cytometry analysis of the MM cell population.

### Cell cycle analysis

Tumors were removed from the primary implanted bones of SCID-rab mice with two contralaterally implanted bones. Tumor tissues were minced and supernatant collected, washed in PBS and fixed in cold 70% ethanol. After removing residual ethanol, propidium iodide (10 μl of 50 μg/ml; BD Biosciences), RNase A (50 μl of 1 mg/ml; Sigma-Aldrich, St Louis, MO, USA) and 0.1% BSA in PBS (200 μl) were added to the cells. After 30 min of incubation in the dark, cell cycle analysis was performed on the FACSCalibur (BD Biosciences) in the Flow Cytometry Core Facility at the UAMS.

### Scid-rab mouse model

SCID mice were obtained from Harlan Laboratories (Indianapolis, IN, USA). SCID-rab mice were constructed as previously described^20^ and were used for *in vivo* metastasis studies and *in vivo* BrdU assays. Briefly, SCID-rab mice were constructed with contralaterally implanted pieces of rabbit femoral bone. Four weeks after implantation, 3.5 × 10^6^ luciferase/enhanced green fluorescent protein-expressing INA6 cells infected with BTK-KD or scrambled control shRNA were directly injected into the implanted rabbit bone on the dorsal right side. Mice were monitored for MM progression by measuring *in vivo* bioluminescence (IVIS Imaging system; PerkinElmer, Waltham, MA, USA) 2 and 5 weeks after MM engraftment; D-luciferin potassium salt (200 μl of 15 mg/ml in PBS; PerkinElmer) was intraperitoneally injected immediately before live-animal imaging. Enzyme-linked immunosorbent assays were used to measure circulating levels of human immunoglobulins^22^ and circulating syndecan-1 (Human Syndecan-1 DuoSet; R&D Systems). Alternate time points were chosen for live-animal imaging and serum collection in order to mitigate stress to the mice. To evaluate metastasis of MM cells to the secondary bones, mice were killed after 10 weeks, mainly due to tumor end point at the primary site. The secondary implanted bone was extracted, crunched in 2 ml PBS and exposed to D-luciferin potassium salt to measure bioluminescence with IVIS imaging. Primary tumors were extracted and cells used to analyze cell cycle and gene expression.

### Global gene expression profiling

CD138-positive MM plasma cells from patients with MM were immunomagnetically selected using autoMACs automated separation system (Miltenyi-Biotec, San Diego, CA, USA) and analyzed with global gene expression profiling (GEP), using U133 Plus 2.0 microarray (Affymetrix, Santa Clara, CA, USA) as described;^7,23^ data were deposited in the NIH Gene Expression Omnibus, accession number GSE2658. Signal intensities were preprocessed and normalized with GCOS1.1 software (Affymetrix).

INA6 MM cells stably infected with scrambled (control) and BTK shRNA were subjected to GEP analyses after their engraftment in SCID-rab mice (three mice/group). Probesets differentially expressed between control and BTK-KD cells were selected based on *P*⩽0.05 statistical significance, 1000 mean signal cutoff of probesets that were overexpressed (in BTK-KD) or underexpressed (in control cells), and at least twofold difference in mean signal between the two groups. Identical analyses were performed using INA6 cells (BTK-KD and control) from cell culture (three culture time points/group) before engraftment in SCID-rab mice.

GEP analysis of primary MM cells before and after coculture with osteoclasts was described elsewhere.^24^

### Statistical analyses

All values are expressed as mean±s.e. of the mean, unless indicated otherwise. Student's *t*-test or paired *t*-test was used to analyze BTK expression and the effect of BTK knockdown on *in vitro* MM cell migration and proliferation and *in vivo* MM cell growth.

## Results

BTK knockdown in INA6 cells reduces *in vitro* clonogenicity potential but has no effect on short-term growth, and promotes growth in coculture with osteoclasts

BTK expression was assessed in shRNA-infected INA6 cells and in primary MM cells. BTK protein was markedly reduced in INA6 MM cells stably infected with lentiviral particles containing BTK shRNA (hereafter BTK-KD cells), as assessed by western blots (Supplementary Figure S1A); *BTK* gene expression was 96% lower in BTK-KD cells than in cells infected with control scrambled shRNA, and expression remained low after cells were engrafted in the SCID-rab mouse model for MM (Supplementary Figures S1B and C). *BTK* expression was lower in primary MM cells (*n*=8 patients) cocultured with osteoclasts than in control precultured MM cells (Supplementary Figure S1D). *BTK* expression remained profoundly lower than scrambled control INA6 cells after coculture with osteoclasts (Supplementary Figure S1E).

**Figure 1:**
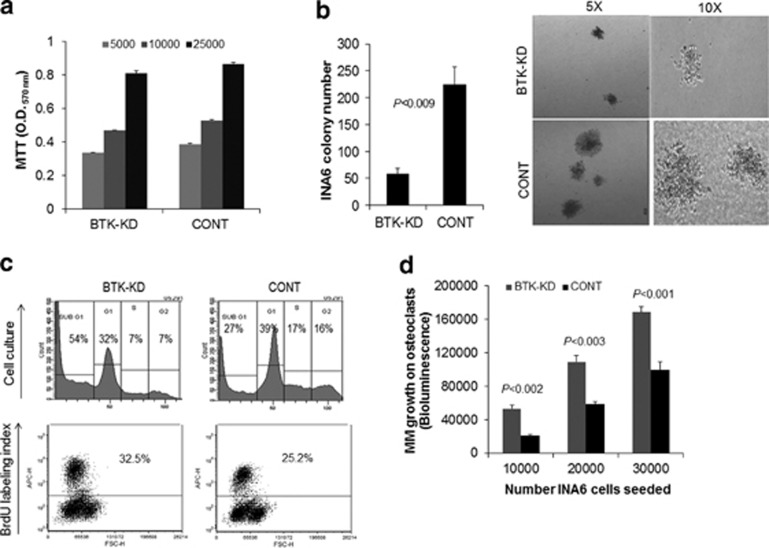
BTK knockdown in INA6 cells reduces *in vitro* clonogenicity but has no effect on short-term growth and promotes growth in coculture with osteoclasts. (**a**) After 3 days of culture, *in vitro* growth of INA6 cells infected with BTK-KD or scrambled control (CONT) shRNA was assessed with MTT assays, which reflect the overall number of viable cells. Cells were plated at three densities (5 × 10^3^, 10 × 10^3^ and 25 × 10^3^ cells/well). (**b**) Left panel, clonogenicity of BTK-KD and CONT cells assessed with 14-day clonogenic assays. Right panel, Representative photographs of typical colonies of BTK-KD and CONT cells at × 5 and × 10 original microscopic magnifications. The clonogenic assay was repeated three times. (**c**) Upper panel: flow cytometry cell cycle analysis of BTK-KD and CONT cells taken from standard cell culture. Lower panel: flow cytometry analysis of BrdU incorporation by BTK-KD and CONT cells taken from standard cell culture. (**d**) Growth of luciferase-expressing BTK-KD or CONT INA6 cells on osteoclasts, measured by bioluminescence. Cells were plated at three MM densities (1 × 10^4^, 2 × 10^4^ and 3 × 10^4^ cells/well) on top of mature osteoclasts and MM growth measured after 3 days.

Effects of BTK knockdown on cell growth and proliferation were evaluated in BTK-KD INA6 cells. Short-term growth in standard culture conditions was similar in BTK-KD cells and those infected with scrambled control (Figure 1a), but 2-week clonogenic assay revealed that BTK-KD cells formed fewer colonies than the control cells (Figure 1b). Flow cytometry analysis showed that BTK-KD cells in their standard culture conditions had a higher fraction in sub-G1 phase and lower fractions in S and G2 phases than did control cells (Figure 1c). Because most sub-G1 cells have fractional DNA content, they are considered apoptotic, but cells in S and G2 phases are considered proliferative.^10,25^ Nevertheless, in clonogenic and MTT assays, we repeatedly used BTK-KD or Control INA6 cells from the cell culture stage with similar viability as determined by Trypan blue cell exclusion method. Because the short-term growth of BTK-KD and control cells was the same, we speculated that BTK-KD cells have a higher *in vitro* proliferative rate but rapidly undergo apoptosis, but control cells do not. As expected, BrdU labeling index showed marginally higher proportions of proliferating BTK-KD cells than of control cells (Figure 1c). In contrast, in coculture with osteoclasts, BTK-KD cells had a higher growth rate than control cells (Figure 1d). Taken together, although overall short-term *in vitro* growth (MTT assay) is similar between the two cell types due to contrasting effects of BTK knockdown on cell proliferation and apoptosis, long-term *in vitro* clonogenicity potential was impaired due to their higher apoptosis rate and lack of supportive microenvironment. In contrast, higher survival rate of BTK-KD cells in coculture with the supportive osteoclasts resulted in increased growth due to their higher proliferation rate.

### BTK knockdown promotes *in vivo* growth of INA6 cells but suppresses metastasis

**Figure 2:**
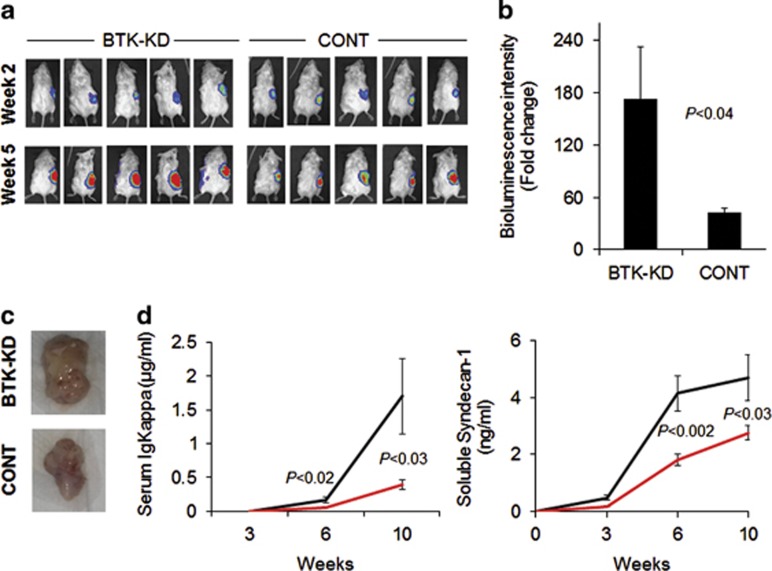
BTK knockdown in INA6 MM cells promotes their growth in SCID-rab mice. (**a**) Live-animal imaging of representative hosts (five mice in each group) 2 and 5 weeks after luciferase-expressing INA6 MM cells infected with BTK-KD or control (CONT) shRNA were engrafted into the implanted rabbit bone. (**b**) Changes in bioluminescence intensity (fold change between 2 and 5 weeks post engraftment) from the primary implanted bones engrafted with BTK-KD or CONT cells (*P*<0.04). (**c**) Representative tumors from primary implanted bones of SCID-rab mice 10 weeks after engraftment of BTK-KD or CONT cells. (**d**) Analysis of MM burden of SCID-rab mice determined by measurements of circulating kappa light chain immunoglobulin (left) and soluble syndecan-1 (right) in sera of mice 3, 6 and 10 weeks after engraftment of BTK-KD (*n*=13; black line) or CONT (*n*=12; red line) INA6 cells.

Because *in vivo* pharmacologic inhibition of BTK activity in MM cells and BM microenvironment cells may indirectly affect MM growth, it was important to investigate whether BTK knockdown in MM cells affects their *in vivo* growth and metastasis. The SCID-rab mouse model was used for the *in vivo* study, with two rabbit bones contralaterally implanted in each SCID mouse; one of the two bones was injected with INA6 cells infected with enhanced green fluorescent protein/luciferase (for tracking cells and monitoring tumor burden) and with shRNA (scrambled control or BTK-KD). In this model, MM cells are capable of metastasizing to the second implanted bone but not to any murine organs.^20^ Some MM cells, such as INA6 cells, grow also on the outer surface of the injected (that is, primary) implanted bone and can be detected in the secondary implanted bone after a relatively long period of time (>10 weeks).^20,26^ Live-animal imaging 2 and 5 weeks after MM cell engraftment revealed tumor growth in the primary implanted bone was 4.1±1.4-fold (*P*<0.04) higher in BTK-KD cells than in control cells (Figures 2a and b). This phenomenon also was visible in photographs of the primary tumors at the end of the experiment (10 weeks after engraftment; Figure 2c). Overall, MM burden at the end of the experiment was higher in the BTK-KD group than in the control group, based on measurements of circulating human immunoglobulins (4.3-fold higher; *P*<0.03) and soluble syndecan-1 levels (2.3-fold higher; *P*<0.03; Figure 2d).

**Figure 3:**
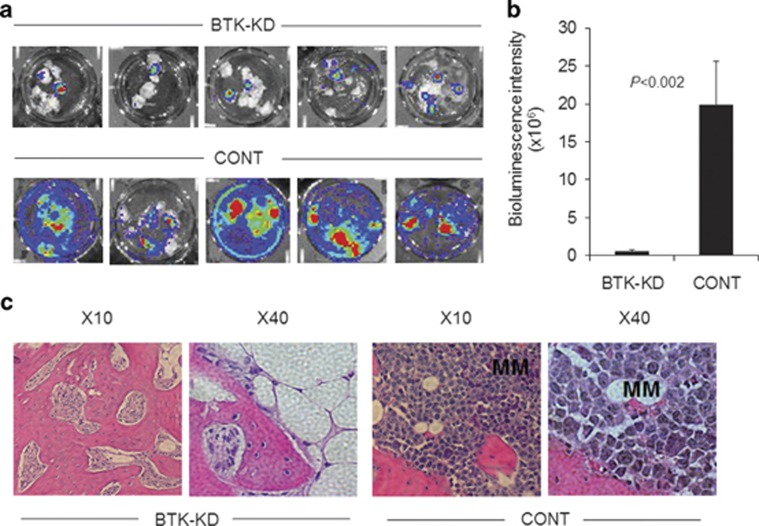
BTK is indispensable for MM cell metastasis to a new BM niche. (**a**) Primary bones of SCID-rab mice (five representative mice in each group, two bones contralaterally implanted in each mouse) were engrafted with luciferase-expressing INA6 MM cells infected with BTK-KD or scrambled control (CONT) shRNA. Ten weeks after engraftment, *ex vivo* bioluminescence of secondary implanted bones was analyzed. Secondary bones were each cut into eight pieces, placed in six-well plates and then immediately subjected to bioluminescence analysis. (**b**) Quantification of bioluminescence intensity in secondary bones from the BTK-KD (*n*=13) and CONT (*n*=12) groups (*P*<0.002). (**c**) Micrographs showing hematoxylin and eosin staining of sections of secondary bones, demonstrating high infiltration of MM cells (clusters of darkly stained cells) in the CONT group but not in the BTK-KD group. Numbers above the images indicate original magnifications.

By 5 weeks after MM cell engraftment, live-animal animal imaging was not informative because of the high tumor burden, so *ex vivo* bioluminescence analysis and histological examination were used at the end of the experiment to assess dissemination of MM cells to murine organs and to the secondary implanted bone. MM cells were not detected in any murine organs. Although overall MM burden was higher in the BTK-KD group, metastasis to the secondary implanted bones was 37±11-fold (*P*<0.002) lower in the BTK-KD group than in the control group (Figures 3a and b). Histological analysis of bone sections confirmed less MM cell infiltration in the secondary bones of the BTK-KD group than of the control group (Figure 3c). Taken together, these data indicate that BTK knockdown in MM cells promotes their growth in a supportive *in vivo* microenvironment but suppresses their ability to metastasize to new BM niches.

### BTK knockdown contrastingly affects survival and proliferation of INA6 MM cells *in vitro* and *in vivo*

**Figure 4:**
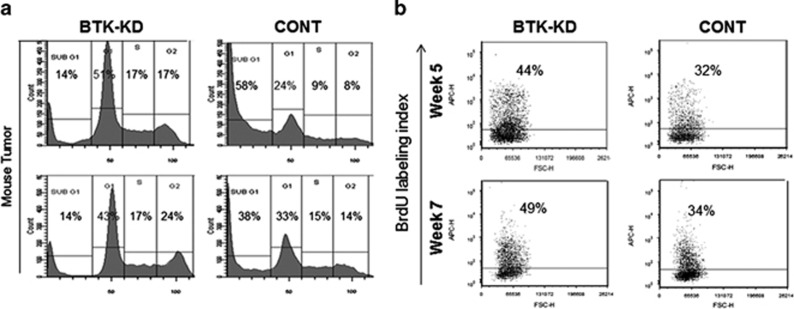
BTK inhibition results in a higher proportion of cells in the G2+M cell cycle phase and proliferation in MM cells within the BM microenvironment. (**a**) INA6 MM cells infected with BTK-KD or scrambled control (CONT) shRNA were extracted after 10 weeks from implanted bones of SCID-rab mice; cell cycle was analyzed with flow cytometry. (**b**) BTK-KD and CONT cells were extracted from implanted bones of SCID-rab mice 5 and 7 weeks after engraftment; BrdU incorporation was analyzed with flow cytometry.

The unexpected findings indicating that BTK inhibition leads to a higher growth rate for MM cells *in vivo* led us to investigate cell cycle and proliferation rates in response to BTK knockdown in SCID-rab mice. MM cells were extracted from the tumors in SCID-rab implanted bones; those engrafted with BTK-KD cells had a lower fraction in the sub-G1 phase (apoptotic cells) and higher fractions in the other cell cycle phases than did those engrafted with cells infected with control shRNA (Figure 4a). This sharply contrasted with observed effects in cultured cells (Figure 1c). *In vivo* BrdU labeling index showed a higher proportion of proliferating BTK-KD cells than of control cells (Figure 4b). These findings suggest that, in the *in vivo* BM microenvironment, lower BTK activity can result in protecting MM cells from spontaneous apoptosis and in directly increasing the proliferative MM cell population.

### BTK knockdown in INA6 cells reduces adhesion and migration and alters expression of factors associated with adhesion, migration and growth

**Figure 5:**
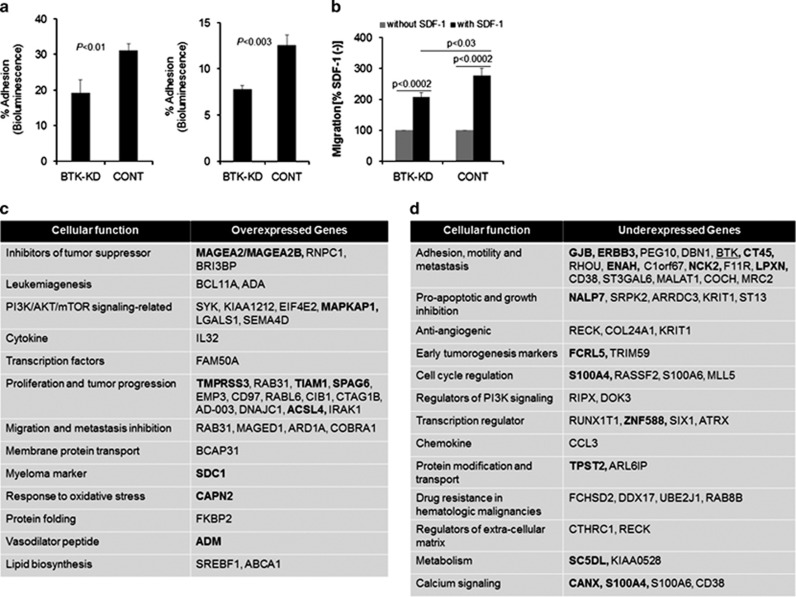
BTK knockdown in MM cells reduces their adhesion, impairs their migration and alters their expression of genes associated with adhesion, migration and growth. (**a**) Bioluminescence analysis of BTK-KD and CONT cells adhering to fibronectin-coated plates alone (left) or with MSCs (right), expressed as percent adherent cells from total cells. (**b**) Migration of BTK-KD and CONT cells toward SDF-1 (30 nM), expressed as percent changes compared with spontaneous migration without SDF-1. (**c**, **d**) Selected overexpressed (high) or underexpressed (low) genes in BTK-KD tumor cells recovered from SCID-rab mice (see Supplmentary Table S1 for the complete list of genes). Bolded gene symbols indicate their expression pattern was originally present in BTK-KD cells cultured in their standard *in vitro* condition (see Supplmentary Table S2 for the complete list of genes).

To shed light onto mechanisms mediating reduced metastasis and increased proliferation that result from BTK inhibition, we analyzed cell adhesion, migration and GEP of INA6 MM cells infected with scrambled control shRNA or BTK-KD. The cultured BTK-KD cells adhered to fibronectin 38±6% (*P*<0.01) less than did control cells, and they adhered to MSCs plus fibronectin 38±3% (*P*<0.003) less than did control cells (Figure 5a). Because the SDF-1/CXCR4 axis is important for homing of MM cells to bone, we investigated whether BTK inhibition affected migration of MM cells toward SDF-1 gradients. BTK knockdown did not reduce cell surface levels of CXCR4 (data not shown) but significantly reduced the ability of INA6 cells to migrate toward SDF-1 (Figure 5b). These data suggest that BTK knockdown reduced migration as well as adhesion of INA6 MM cells.

GEP analysis was performed on INA6 cells infected with scrambled control shRNA or BTK-KD after being recovered from the primary bones implanted in SCID-rab mice. Expression of 109 probesets was more than twofold higher in BTK-KD cells than in control cells, and expression of 110 probesets was more than 0.5-fold lower in BTK-KD cells than in control cells (Supplementary Table S1). Similar analyses on the control and BTK-KD cells grown in their standard *in vitro* conditions revealed 40 overexpressed and 47 underexpressed probe sets in BTK-KD cells (Supplementary Table S2). We focused our analyses on genes differentially expressed in the *in vivo* experiment. Selected upregulated or downregulated genes have been listed based on their cellular function (Figures 5c and d). *BTK* was among the most underexpressed genes in BTK-KD cells, confirming our quantitative reverse transcriptase-PCR and western blot analyses. Among the genes most upregulated in BTK-KD cells were several factors associated with lymphoid malignancies, including *BCL11A*, *SYK* and *TIAM1*, and tumor- and testis-related antigens associated with proliferation, such as *RAB31*, *MAGEA2* and *SPAG6*. Several genes related to PI3K/AKT/mammalian target of rapamycin (mTOR) signaling were upregulated such as *LGALS1*, *EIF4E2* and *SEMA4D* suggesting increased cell activity associated with proliferation. We confirmed the increased expression of mTOR protein and its phosphorylation state in BTK-KD cells compared with control INA6 cells (Supplementary Figure S2). In agreement with higher tumor burden by BTK-KD MM cells *in vivo* was the overexpression of MM-associated markers such as *SDC1* and *CLK2* and heparanase target gene, *PLXNC1*.^27,28^ Additional upregulated genes, such as *ADA*, negatively regulate leukocyte migration (Figure 5c). The list of underexpressed genes includes several factors associated with cell adhesion, motility or cytoskeleton rearrangement, such as *LPXN*, *DBN1*, *F11R, ST3GAL6, RHOU* and *CT45.* Growth inhibitory genes such as *NALP7*, which inhibits IL-1β production, and metastasis-associated genes such as *S100A4* are underexpressed^29,30^ (Figure 5d). Together, these findings suggest that BTK regulates the expression of distinct factors mediating MM cell adhesion, metastasis and proliferation.

### Intratumoral heterogeneity of BTK and CXCR4 expression in clinical MM

**Figure 6:**
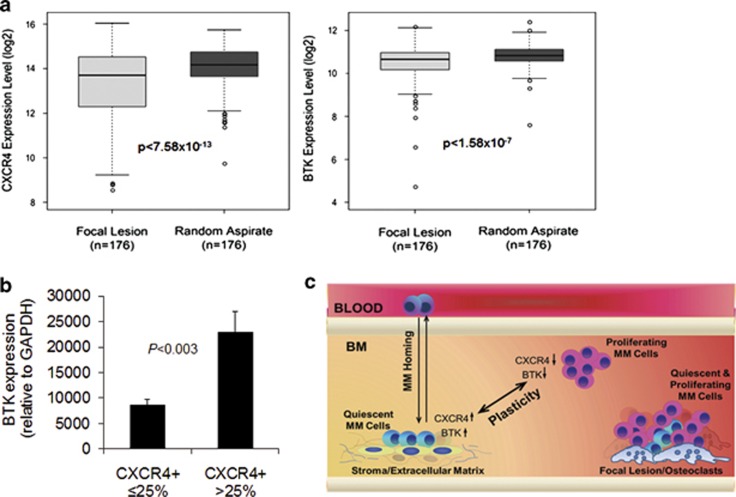
Expression of *CXCR4* and *BTK* is linked, and both genes are underexpressed in MM cells from focal lesions compared with random-site aspirates from the same patients. (**a**) Expression of *CXCR4* and *BTK* was analyzed from cells of paired samples of focal lesions and random BM. Distributions of the differences for each pairwise comparison of each gene (*CXCR4*, *P*<7.58 × 10^−13^; *BTK*, *P*<1.58 × 10^−7^) are presented as box-and-whisker plots. Lower and upper edges of the boxes correspond to the first and third quartiles, respectively. The thicker bars in the middle represent the median, and the whiskers extend to the minimum and maximum values. (**b**) Plasma cells from random BM aspirates of patients with MM were analyzed for expression of BTK; samples were stratified according to the proportion of cells (⩽25% or >25%) with CXCR4 on their surfaces. (**c**) A model of intratumoral heterogeneity in myelomatous bone. Expression of BTK and CXCR4 in MM cells growing in interstitial BM is variable and is affected by interactions with matrix proteins and stromal cells. Prolonged persistence of MM cells in focal lesions results in mixed populations. BTK and CXCR4 expression impacts adherence and proliferative and metastatic behavior of these cells. BTK is essential for homing of MM cells from the circulation to the bone.

We previously demonstrated the association between *BTK* expression and cell surface levels of CXCR4 in primary MM cells and that *BTK* expression is more profound in CXCR4^+^ than in CXCR4^–^ MM cells sorted from the same patient sample.^16^ To further examine the intraclonal heterogeneity of MM cells with regard to these two factors, we analyzed the expression of *BTK* and *CXCR4* in CD138-selected MM cells obtained from 176 paired samples—random-site BM aspirates and computed tomography-guided fine-needle aspirates of focal lesions defined by magnetic resonance imaging from the same patients. Expression of *BTK* (*P*<1 × 10^−7^) and *CXCR4* (*P*<7 × 10^−13^) was significantly lower in samples from focal lesions than in those from random BM sites (Figure 6a). Furthermore, in samples from random-site BM aspirates that contained lower proportions (<25%) of MM cells with cell surface CXCR4, *BTK* expression was significantly lower than in samples with higher proportions (>25%) of MM cells with cell surface CXCR4 (Figure 6b). Thus, the differential expression of *BTK* and *CXCR4* in focal lesions and interstitial marrow reflects intraclonal heterogeneity and points to the potential roles of distinct microenvironmental sites in regulating expression of these factors in MM cells.

## Discussion

We demonstrated that, in INA6 MM cells growing in the supportive *in vivo* SCID-rab model for MM, BTK knockdown promoted MM cell growth but inhibited metastasis to new BM niches. The *in vitro* clonogenicity potential of BTK-KD cells was reduced, but, in coculture with the supportive osteoclasts, these cells had a higher growth rate than did control cells. Our study also revealed that the expression of *BTK* and *CXCR4* is intratumorally heterogeneous in clinical MM samples from focal lesions and interstitial marrow of the myelomatous bone, suggesting that BTK is involved in determining proliferative, quiescent or metastatic phenotypes of MM cells (Figure 6c).

Although it is recognized that BTK has an important role in MM cell adhesion, migration and homing to bone,^16,17^ the involvement of this tyrosine kinase in MM cell proliferation and growth requires further clarification. A study using a genome-scale small-interfering RNAs screening in MM cells revealed enhanced growth of MM cells by BTK small-interfering RNAs, supporting our findings.^31^ Recently, Tai *et al.*^17^ demonstrated that treatment with ibrutinib (BTK inhibitor) or shRNA-mediated transient inhibition of BTK is directly cytotoxic to MM cell lines *in vitro*. They also showed that ibrutinib treatment inhibits the clonogenic potential of stem-like cells sorted from MM cell lines or MM patients, and it inhibits growth of INA6 cells in the SCID-hu model.^17^ Further, relatively high concentrations of BTK inhibitors have been shown to enhance the cytotoxic effects of bortezomib and lenalidomide on MM cell lines *in vitro*.^32^ In contrast, we recently reported that short-term *in vitro* growth of INA6 cells was not profoundly affected by BTK inhibitor LFM-A13 or from stable knockdown of BTK, whereas another study using BTK inhibitor CC-292 showed moderate inhibitory effects on INA6 cells but not on other MM cell lines.^16,19^ In the present study, clonogenicity of BTK-KD cells was lower than control cells, but their overall growth in coculture with supportive osteoclasts was enhanced and their growth and proliferation in the SCID-rab model were higher than control cells. This suggests that, in certain microenvironmental conditions, BTK inhibition in MM cells provides superior survival conditions that augment MM cell proliferative potential and facilitate MM propagation. Indeed, although BTK-KD INA6 cells cultured *in vitro* had a higher proportion of apoptotic cells (defined by sub-G1 cell cycle phase), those recovered after engraftment in SCID-rab mice had a significantly lower proportion of apoptotic cells, a higher proportion of proliferative cells (in S+G2 cell cycle phases),^10^ and increased BrdU labeling index. *In vitro*, BTK-KD cells had increased growth over osteoclasts suggesting that the reversed phenomenon in cell cycle observed *in vivo* could be due to the supportive effect of myelomatous BM microenvironment. However, the proliferation of BTK-expressing control INA6 cells *in vivo* could be regulated through their adhesion to BM stroma. It is noteworthy that most stroma-independent MM cell lines, which typically exhibit phenotypes of very high proliferation and survival, express extremely low levels of BTK^16^ and their growth in immunodeficient animal models is often not restricted to the bone. Rather than inconsistency, our findings suggest that BTK expression and function are distinct in MM cells that are signaled by the microenvironment to either proliferate or to adhere and become quiescent.

Because BTK is expressed in MM cells and various BM cells, the experimental settings are crucial for understanding the role of this factor in MM. Our *in vivo* experimental setting using BTK knockdown exclusively in MM cells is different from the *in vivo* study using systemic administration of BTK inhibitors in MM-bearing SCID-hu mice.^16,17^ In the SCID-hu model, in which a human fetal bone is implanted in the SCID mouse, human hematopoiesis in the implanted bone is minimal, and reduced growth of MM cells resulting from ibrutinib treatment could be due to marked inhibition of osteoclast activity, which has been reported for BTK inhibitors^16,17,19^ and other agents.^26^ In the experiment reported here, however, BTK inhibition was limited to the engrafted MM cells and remained low throughout the course of the *in vivo* study, so any effects on the microenvironment would be indirect.

Evidence supports the notion that BTK mediates the behavior of subpopulations of MM cells within the BM microenvironment. We recently demonstrated that variable levels of CXCR4 are present on cell surfaces of different subpopulations of MM cells and that cell surface levels of CXCR4 highly correlate with *BTK* expression in primary MM cells.^16^ Expression of BTK is higher in MM cells adherent to fibronectin or to stromal cells than in nonadherent cells.^17,33^ The current work showed that the levels of CXCR4 and BTK were relatively lower in MM cells residing within focal lesions than in those within interstitial marrow and in primary MM cells after coculture with osteoclasts. Furthermore, in MM cells from random BM aspirates, the proportion of MM cells with cell surface CXCR4 was positively associated with *BTK* expression levels. Lower CXCR4 gene expression in MM cells has been associated with poor outcome^34^ and resistance to bortezomib^35^ in patients with MM. BTK-KD INA6 cells exhibited reduced adhesion and migration, lower metastatic behavior and higher proliferation. These findings point to a dynamic plasticity mechanism,^36^ whereby MM cell adherence and quiescence is associated with higher levels of CXCR4 and BTK, whereas MM cell proliferation is associated with lower levels of CXCR4 and BTK.

Results of GEP analyses of BTK-KD INA6 cells further support the role of BTK in MM cell adhesion, metastasis and growth. Several of the most downregulated genes (for example, *ST3GAL6*, *CT45*, *LPXN, RHOU*) have been implicated with cellular adhesion and motility, whereas many upregulated genes are associated with activation of PI3K/AKT/mTOR signaling (for example, *LGALS1*, *EIF4E2, MAPKAP1, SEMA4D*) and cell proliferation (for example, *BCL11A*, *RAB31*).^37, 38, 39^ In BTK-KD INA6 cells, increased protein expression and phosphorylation of mTOR suggests that the loss of BTK and subsequent adhesion to stroma may increase proliferative potential of MM cells. For instance, overexpression of *RAB31* converts breast cancer cells from an invasive phenotype to a proliferative one.^40^ Also, knockdown of CT45 genes in various tumor cells, including MM cells, results in diminished adhesion and migration,^41^ consistent with our findings in BTK-KD MM cells. *COL24A1* has antiangiogenic function in MM and its downregulation suggests that BTK-KD MM tumors are well equipped with angiogenic vessels for enhanced growth.^42^ These observations suggest that adhesive signals from MM cells could permit BM microenvironment to dictate tumor growth partly through regulation of constitutively active RAS/PI3K/mTOR pathway. Further, our GEP analysis provides mechanistic clues and implicates potential target genes of BTK.

In summary, the knockdown of BTK in MM cells resulted in reduced adhesion to stroma, impaired migration, increased *in vivo* MM cell proliferation and growth and suppressed metastasis to a new BM niche. Our observation that *BTK* and *CXCR4* expression are lower in focal lesions and our report that levels of these two factors are linked in MM cells^16^ support the notion that diverse subpopulations mediate MM cell proliferation, adhesion and metastasis. Our findings contribute to understanding the dynamics and complexity of intratumoral heterogeneity and plasticity, which are now acknowledged in MM^43^ and other cancers^44^ as a means to explain tumor behavior and stages of disease progression. Although our conclusions are based on a single MM line, the findings of intratumoral heterogeneity and differential behavior of MM subpopulations are clinically relevant. Our future work will determine whether additional phenotypical and genomic changes differentiate MM cells and the microenvironment in focal lesion from their interstitial BM counterparts. Identification of signaling pathways downstream of BTK that mediate MM cell metastasis and the exact role of BTK in patient MM cell proliferation and growth also pending additional investigations. Clinically, BTK inhibition in MM has potential in improving patient survival mainly through management of bone disease and metastasis to new BM niches. Novel BTK inhibitors may be helpful in reducing MM tumor burden partly and indirectly through reduction of osteolytic activity in bone microenvironment, and directly when combined with clinical cell proliferation inhibitors such as certain alkylating agents and proteasome inhibitors.

## Supplementary Material

Supplementary Figure legend

Supplementary Table1
