# Supplementary material for: Role of Bruton's tyrosine kinase (BTK) in growth and metastasis of INA6 myeloma cells
Source: Blood Cancer J. 2014 Aug 1;4(8):e234–. doi: 10.1038/bcj.2014.54 (PMC4219470; doi:10.1038/bcj.2014.54)
Supplement: Supplementary Figure legend [file bcj201454x1.doc]

**Bam et al. Supplementary Figure S1**

**
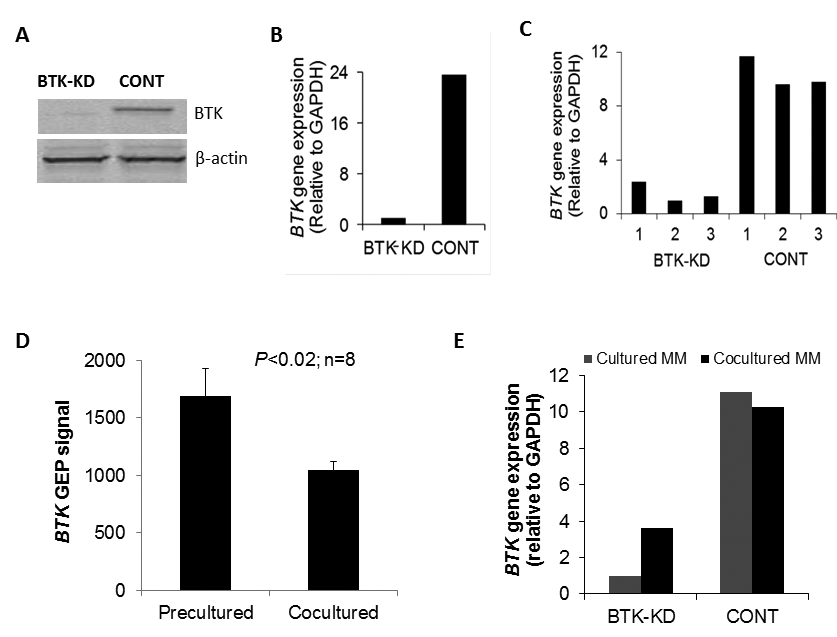
**

**Supplementary Figure S1. BTK expression in INA6 MM cell line and primary MM cells.**

A, Immunoblot of BTK and β-actin in INA6 cells stably infected with *BTK*-targeted shRNA (BTK-KD) or scrambled control shRNA (CONT). B and C, BTK qRT-PCR analysis of BTK-KD and CONT cells perform during their *in vitro* culture (B) and after establishment of growth in three representative SCID-rab mice (C). D, GEP analysis of BTK gene expression in primary MM cells before (Precultured) or after (Cocultured) coculture with osteoclasts. E, BTK gene expression in BTK-KD and CONT cells cultured in their standard conditions (cultured MM) or cocultured with osteoclasts (cocultured MM).
