# Supplementary material for: Role of Bruton's tyrosine kinase (BTK) in growth and metastasis of INA6 myeloma cells
Source: Blood Cancer J. 2014 Aug 1;4(8):e234–. doi: 10.1038/bcj.2014.54 (PMC4219470; doi:10.1038/bcj.2014.54)
Supplement: Supplementary Figure legend [file bcj201454x2.doc]

**Bam et al. Supplementary Figure S2**


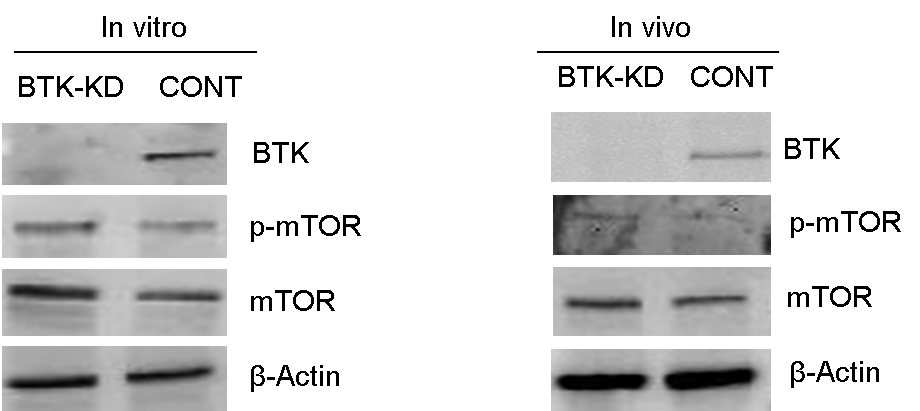


**Supplementary Figure S2. mTOR signal in BTK-KD cells and CONT INA6 cells from standard cell culture media or mouse tumor.**

Immunoblot of INA6 BTK-KD and Control (CONT) cells grown in standard *in vitro* conditions or those recovered from the primary bones implanted in SCID-rab mice show higher protein expression and S2448 phosphorylation state of mTOR. Although mTOR signaling is increased in BTK-KD cells both in *in vitro* and *in vivo* conditions, it has substantial implications in tumor growth *in vivo*.
